# A Novel Strategy for Selective Thyroid Hormone Determination Based on an Electrochemical Biosensor with Graphene Nanocomposite

**DOI:** 10.3390/s23020602

**Published:** 2023-01-05

**Authors:** Sylwia Baluta, Marta Romaniec, Kinga Halicka-Stępień, Michalina Alicka, Aleksandra Pieła, Katarzyna Pala, Joanna Cabaj

**Affiliations:** 1 Faculty of Chemistry, Wrocław University of Science and Technology, Wybrzeże Wyspiańskiego 27, 50-370 Wrocław, Poland; 2 Food4Future Technologies Sp. z o.o., ul. Tarasa Szewczenki 24, 51-351 Wrocław, Poland

**Keywords:** antibodies, nanomaterials, laccase, thyroid hormones, voltammetry

## Abstract

This article presents a novel and selective electrochemical bioassay with antibody and laccase for the determination of free thyroid hormone (free triiodothyronine, fT3). The biosensor was based on a glassy carbon electrode modified with a Fe_3_O_4_@graphene nanocomposite with semiconducting properties, an antibody (anti-PDIA3) with high affinity for fT3, and laccase, which was responsible for catalyzing the redox reaction of fT3. The electrode modification procedure was investigated using a cyclic voltammetry technique, based on the response of the peak current after modifications. All characteristic working parameters of the developed biosensor were analyzed using differential pulse voltammetry. Obtained experimental results showed that the biosensor revealed a sensitive response to fT3 in a concentration range of 10–200 µM, a detection limit equal to 27 nM, and a limit of quantification equal to 45.9 nM. Additionally, the constructed biosensor was selective towards fT3, even in the presence of interference substances: ascorbic acid, tyrosine, and levothyroxine, and was applied for the analysis of fT3 in synthetic serum samples with excellent recovery results. The designed biosensor also exhibited good stability and can find application in future medical diagnostics.

## 1. Introduction

**Figure 1:**
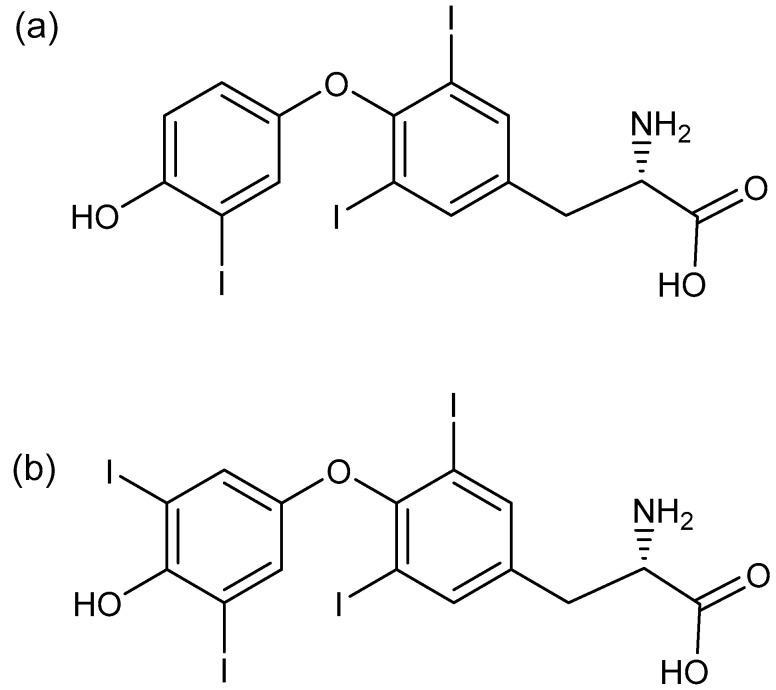
Chemical structure of (**a**) fT3 and (**b**) T4 hormones.

Proper functioning of thyroid hormones has a great impact on the human organism. Iodinated amino acids, 3,3′,5,5′-tetraiodo-l-thyronine (T4), and 3,3′,5-triiodo-l-thyronine, or triiodothyronine (free T3, fT3) (Figure 1) are hormones produced and secreted by the thyroid gland [1]. Free T3 in healthy individuals is the active form of a thyroid hormone and plays a vital role as it promotes the transition from neonatal to autonomous life [2], maintenance of physiological function, energy homeostasis, and is necessary for normal development. For instance, fT3 is involved in skeletal growth [3], lung maturation [4], and evolution of a few brain regions [5], but also Ng et al. suggest its involvement in sensory development [2]. Thyroid dysfunction is a common clinical problem causing many serious disorders, as well as affecting a large part of society, very often women of reproductive age, due to the fact that unbound (or free) fT3 and T4 influence the synthesis and release of thyroid stimulating hormone (TSH), causing the levels of the circulating thyroid hormones to be over or below the required range [5]. Therefore, an accurate assessment of thyroid function is of great importance for both the diagnosis and monitoring of thyroid diseases [6].

Of late, enormous advances have been made in immunosensors for use in many fields of industry, such as agriculture, food safety, biomedicine, quality control, or environmental analysis [7]. Unfortunately, the literature is still missing descriptions of fT3 immunosensing techniques, as most published research data focus on electrochemical-based biosensors for thyroid stimulating hormone (TSH) or T4 detection. From a medical, analytical point of view, it is extremely important to work also on biosensors that allow the monitoring of fT3, as an indicator of thyroid diseases. Various ways are used to determine the level of fT3, such as radioimmunoassay (RIA), enzyme immunoassay [8], chemiluminescence, or electrochemiluminescence [9], however, they require multiple-step preparation, processing, specific laboratory equipment, are time-consuming, and do not allow continuous fT3 monitoring [6]. For instance, RIA uses a displacing agent, such as 8-anilino-1-naphthalene-sulfonic acid or salicylate, to detach the hormone [10,11,12]. This is why the development of immunosensors is very important from a medical point of view. Medical diagnostics requires fast and sensitive analytical methods which will allow for a rapid test, but also permanent monitoring of important parameters. Biosensors, including microfluidic [13] and electrochemical [14,15], are very promising approaches in this context. Most attention is paid to immunosensors, which use antibodies in the biorecognition part. Immunosensors detect the binding event between an antibody (Ab) and an antigen (Ag) with the formation of a stable Ab–Ag complex. Antibodies, along with enzymes, are among the most widely chosen molecules for the biorecognition layer because of their high specificity and affinity [16]. A combination of a biorecognition element based on antibodies with an electrochemical transducer in biosensors results in a highly specific, sensitive, and fast diagnostic test. The basis of the development of an electrochemical immunosensor characterized by good working parameters, such as linearity, sensitivity, selectivity, and short response time, is the construction of the biorecognition part close to the transducer. In case of electrochemical biosensors, such parameters are achieved by coupling the biorecognition element almost directly onto the electrode (transducer) surface. 

Mainly electrochemical immunosensors have attracted considerable interest due to their advantages, such as simple pretreatment procedure, fast analytical time, low detection limits, low cost, and the possibility of miniaturization [17]. Measurements in a voltammetric immunosensor are based on the application of constant potential at the working electrode, which is in close proximity to the reference electrode, and the current is registered due to electrochemical oxidation or reduction of an investigated electroactive species [18]. However, because most antibodies and antigens are not electroactive [19], the immunosensor utilizes electroactive labels or mediators to achieve an amperometric response. Such indirect (labeled) immunosensor is based on a signal generated from one (or more) markers, thanks to which a sensitive, as well as comprehensive detection is possible. Enzymes, mainly belonging to the class of oxidoreductases, can be used for this purpose [20,21,22]. Oxidoreductases allow higher electrochemical signals to be obtained due to a catalytic reaction of the enzyme labeled as a probe with the detection antibody. Laccase belongs to the multicopper oxidases (it consists of four copper ions in the active center, classified into type-1, type-2, and two type-3 ions) and catalyzes the oxidation of a number of phenolic derivatives and aromatic amines with simultaneous reduction of oxygen to water (O_2_ + 4H^+^ + 4e^−^ → 2H_2_O) [23]. Based on the generally accepted hopping intramolecular electron transfer mechanism for oxygen reduction by laccase [24], the type-1 center is the primary mononuclear Cu center which receives electrons from phenolic derivative and then transfers electrons to the type-2/type-3 redox copper center, and fully reduced trinuclear copper center reacts with dioxygen [25]. So, in general, the oxidation-reduction capacity of this enzyme is imparted by the transfer of electrons that takes place between the type-1 and type-2/type-3 sites, with the reduction of molecular oxygen to water. Moreover, conductive nanomaterials are also very often used to amplify the signal, such as graphene-based nanocomposites. For instance, Fe_3_O_4_, magnetite, nanoparticles (NPs) have become a great focus of interest for a large number of scientific groups all over the world. In the nanorange, magnetite particles indicate superparamagnetic properties [26]. Such nanomaterial is frequently used as a promising electrode modifier due to its unique properties, thanks to which it can improve the electrochemical performance of an electrode (a parameter directly related to the absorption capacity and the conductivity of the modified material)[27]. The Fe_3_O_4_ nanoparticles, as an easy to prepare, non-toxic, with excellent absorption capacity, catalytic properties, and inherent electrical conductivity [28] material, is often a natural choice in electrochemical sensors applications. The deposition of Fe_3_O_4_ nanocomposites onto the electrode surface enhances the electrode area and the rate of electron transfer, improves selectivity and sensitivity, and results in an increased response to noise ratio [29]. In addition, combining Fe_3_O_4_ with other nanomaterial or conductive material, such as graphene, which is very often used in numerous electrochemical approaches [30,31], results in improving adsorption capacity, which makes the composites suitable for the electrochemical detection of small concentrations of important analytes, such as thyroid hormones [32]. Fe_3_O_4_ NPs easily aggregate with different species, such as graphene, which results in lowering their magnetic properties, however, at the same time can increase the biocompatibility properties [33]. Labeled immunosensors have advantages in comparison with direct immunoassays, such as higher sensitivity and less influence of nonspecific adsorption on the signal [34]. 

In addition, in the absence of antigen–antibody interaction, no signal should be observed; however, it is often the case that a slight peak is visible due to the nonspecific binding of the antigen or other proteins to the surface. This type of non-specific absorption also leads to an increase in the background signal and therefore results in a decrease in sensitivity [35]. Hence, it is necessary to use a blocking agent such as bovine serum albumin (BSA) [36]. 

Numerous techniques could be used in such systems, but frequently applied are cyclic voltammetry (CV), differential pulse voltammetry (DPV), and electrochemical impedance spectroscopy (EIS). Wei and co-workers applied the DPV method for immunosensing, where they developed an amperometric immunosensor for chlorpyrifos-methyl (CM), a persistent insecticide, based on an immunogen/platinum doped silica sol–gel film modified screen-printed carbon electrode [37]. Scientists showed that the linear response to CM concentration based on the DPV method was in the range from 0.4 to 20 ng mL^−1^. Moreover, the detection of CM with the described method was also tested in soil and grape samples. The results indicate that these samples matched the reference values well, confirming that the proposed immunosensor can be a promising application for environmental and food analysis. Another electrochemical system was developed by Zang et al. and was focused on CV. Using this method, the authors in their work detected an ofloxacin (OFL), an anti-bacterial drug [38]. An immunosensor was based on a dual signal amplified strategy using a polypyrrole film–Au nanocluster matrix on a glassy carbon electrode (GCE) as a sensor platform and multi-enzyme-antibody functionalized gold nanorod (the authors used horseradish peroxidase (HRP) and horseradish peroxidase-secondary antibody (HRP-Ab2)) as an electrochemical detection label. Described immunosensor for OFL exhibited a sensitive response in the concentration range from 0.08 to 410 ng mL^−1^ with a detection limit of 0.03 ng mL^−1^. Moreover, it exhibits good sensitivity, selectivity, and long-term stability. Singh and co-workers described an immunosensor for 17β-estradiol (E2) detection based on the EIS technique [39]. The immunosensor was constructed by functionalizing the electrode using self-assembled monolayers coupled with a specific monoclonal antibody (mAb) against E2. The authors also described the basic working parameters of the proposed immunosensor: linearity in the range of 5–200 pg mL^−1^, the limit of detection equal to 1 pg mL^−1^ (based on signal to noise equation − S/N = 3) with a short analysis time of 10 min, and stability of 14 days. Additionally, the proposed method meets the regulatory requirements of the European Union (50 pg mL^−1^) or Food and Drug Administration (120 pg mL^−1^) standards.

Herein, we present, for the first time to our knowledge, an electrochemical biosensor for the detection of fT3 by integrating an antibody with laccase for good selectivity and signal amplification. The biosensor was based on three main components: a GCE decorated with a Fe_3_O_4_@graphene nanocomposite as a semiconducting material, an antibody highly specific for fT3, and laccase responsible for catalyzing the redox reaction, recorded during electrochemical measurements. We also demonstrate that laccase can catalyze the oxidation reaction of fT3. We show that the analytical performance of the presented biosensor is acceptable, with the detection limit within the nanomolar range, a wide linear range, good selectivity, and stability. The proposed test is easy to prepare, uses non-toxic reagents, and possesses the possibility of miniaturization.

## 2. Materials and Methods

### 2.1. Chemicals

Laccase (oxygen oxidoreductase, EC 1.10.3.2, from *Trametes versicolor*, ≥0.5 U mg^−1^), 3,3′,5-triiodo-l-thyronine ≥95% (fT3), antibody protein disulfide isomerase family A, member 3 (PDIA3) (Anti-PDIA3) produced in rabbit 0.10 mg mL^−1^ (Ab), ascorbic acid (AA), tyrosine (Tyr), Human Serum from human female AB plasma, USA origin, sterile-filtered, bovine serum albumin (BSA), and Fe_3_O_4_@graphene nanocomposite, 10 mg mL^−1^ dispersion in acetone (Product of USA), were purchased from Sigma-Aldrich Co (Merck company). Citric acid (CA), NaOH, NaH_2_PO_4_, Na_2_HPO_4,_ KH_2_PO_4_, Tris, HCl, CH_3_COONa, CH_3_COOH, NaCl, KCl, and glutaraldehyde (GA) were purchased from POCH (Part of Avantor, Performance Materials, Poland). Levothyroxine (Euthyrox N 75) was manufactured by Merck. All chemicals were of analytical grade and were used without further purification. All buffers were prepared according to generally known, obligatory standards.

### 2.2. Apparatus

All electrochemical measurements were performed using a potentiostat/galvanostat Autolab PGSTAT 128N with NOVA software and typical three-electrode 8 mL cell equipment. Glassy carbon electrode (GCE, diameter 3 mm, produced by BASi, MF-2012 model), modified or unmodified, was adopted as a working electrode, silver/silver chloride electrode was used as a reference electrode, and platinum wire as a counter electrode. The visualization and characterization of nanomaterials based on Fe_3_O_4_@graphene were carried out using a scanning electron microscope (SEM, model JEOL JSM-661OLV) at 16 kV of beam voltage. The nanocomposite was applied directly to the microscope aluminum stubs covered with carbon tape, without sputtering. Observed measurements were averaged.

### 2.3. Fabrication of the Biosensor

The fabrication of the biosensor was a multistep modification process of a glassy carbon electrode. GCE was used for this purpose due to its good electrical conductivity, chemical stability, biocompatibility, workability in a wide potential range, and extremely low gas permeability [40]. First, the GCE was washed with acetone, polished to a smooth surface with diamond powder (diameter 3 µm), completely rinsed with deionized water, and dried at room temperature. A homogeneous suspension of nanocomposite was used to modify the electrode, which is often reached by physical deposition onto the electrode surface. Then, 20 μL of 10 mg mL^−1^ Fe_3_O_4_@graphene nanocomposite was dropped onto the GCE surface and dried at room temperature overnight. Subsequently, hydroxyl groups were introduced to the surface by incubating the electrode in 0.1 M NaOH solution for 15 min at room temperature and drying, which could then be reacted with 10% glutaraldehyde (15 min incubation at room temperature). That in turn allowed the covalent bonding of a specific antibody (Ab) to the modified electrode through an imine linkage between the primary amine group of the antibody and the carbonyl group of the glutaraldehyde (antibody incubation lasted for 2 h at 37 °C). Then, 20 µL of the anti-PDIA3 (Ab, polyclonal, primary antibody) solution (10 µg mL^−1^) was dropped onto the electrode and kept for 1 h (to prevent surface drying during binding) to covalently bind the anti-PDIA3 Ab to the activated surface. Finally, an innovative approach of using both anti-PDIA3 Ab and laccase (2 mg mL^−1^) was used, in which the enzyme was immobilized on the surface of the electrode through physical adsorption (incubation for 2 h at 35 °C). Laccase belongs to the oxidoreductases and was used for the visualization of the redox reaction of fT3. The detection was based on specific interaction between the antibody and fT3, which allowed to direct the analyte to the active site of the enzyme, therefore enabling its oxidation. In addition, to avoid nonspecific binding, bovine serum albumin (10% BSA) was used to block free space and nonspecific binding (incubation for 30 min at room temperature), thus obtaining a biosensor.

The modified electrode was then rinsed with 0.1 M phosphate (pH 7.0) and 0.1 M citric buffers (pH 5.2) for 15 min, then 0.1 M Tris-HCl (pH 7.2, 45 min) and 0.1 M PBS (pH 7.0, 60 min) buffers to wash unbound proteins from the electrode surface.

**Figure 2:**
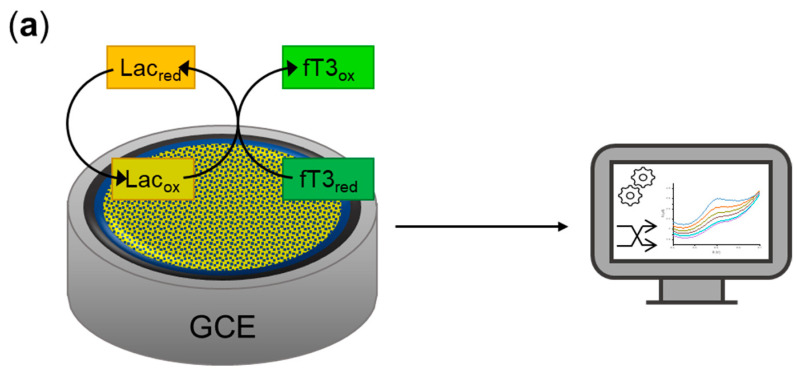

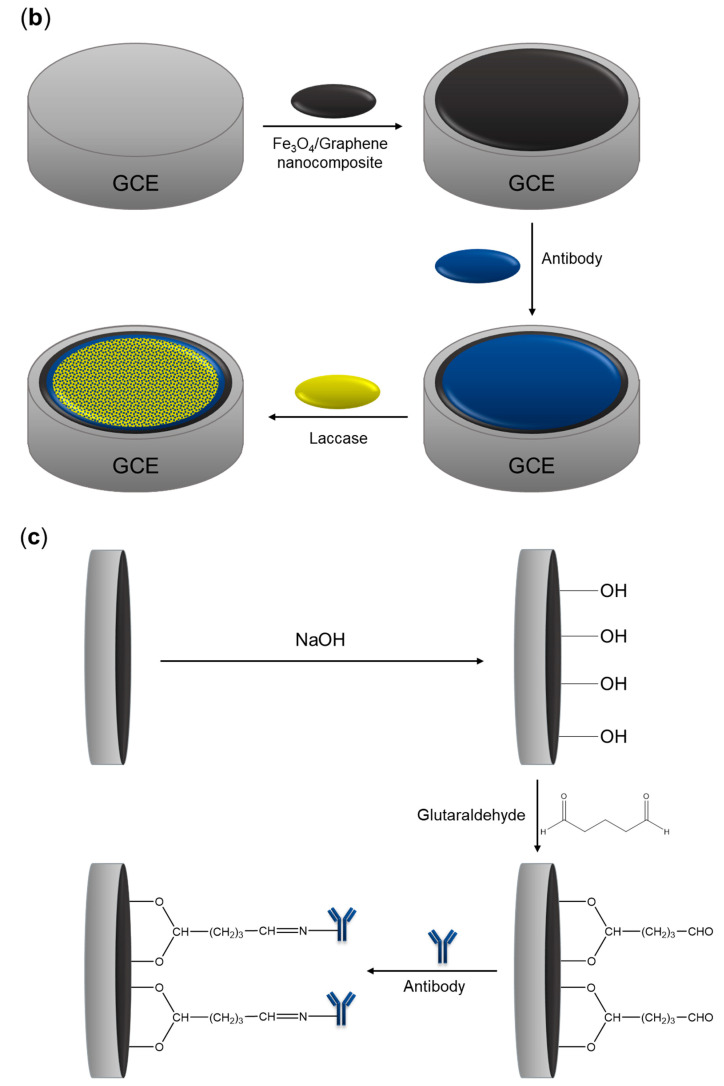
(**a**) Schematic representation of the biosensor’s working principle; (**b**) schematic representation of the main steps of the preparation of the biosensor for the determination of fT3; (**c**) proposed, specified mechanism of the electrode modification with the antibody. GCE—glassy carbon electrode; Lac—laccase.

The detailed construction steps of the biosensor are shown in Figure 2. In the next step, the cyclic voltammetry (CV) analysis was used for the visualization of a successful reaction of reagents and antigen-antibody binding in 200 µM fT3 dissolved in 0.1 M PBS (pH 7.0). The modified GCE/Fe_3_O_4_@graphene/Ab/Lac electrode, prepared according to the described procedure, was stored at 4 °C when not in use.

### 2.4. Electrochemical Procedure of fT3 Analysis

The determination of the thyroid hormone (fT3) was conducted using the electrochemical equipment described in Section 2.2. The cyclic voltammetry (CV) technique was adopted for the redox reaction observation. Measurements were performed in the potential range of −0.2 to 1.3 V vs. Ag/AgCl for 3 cycles each, at a scan rate of 50 mV s^−1^. For the characterization of the working parameters of the biosensor, such as linear range, detection limit, selectivity, and real sample analysis, the differential pulse voltammetry (DPV) technique was used. DPV for a range of concentrations of fT3 was provided in the potential range of 0.3–0.7 V vs. Ag/AgCl and with a step potential of 5 mV, modulation amplitude of 25 mV, modulation time of 0.05 s, and interval time of 0.5 s. To test the ability of the biosensor to work under open-air conditions and at room temperature, as well as for the proper catalytic activity of the enzyme, which requires access to atmospheric oxygen [41], all electrochemical measurements were performed under such conditions. Solutions of fT3 in the concentration range of 10–200 μM were prepared by dissolving fT3 in 0.1 M PBS buffer at pH 7.0. The current response obtained during the analysis was proportional to each given concentration.

### 2.5. Selectivity and Stability Tests

Common and similar in structure to fT3 exemplary species have been tested for possible interference during fT3 measurements. Ascorbic acid (AA), tyrosine (Tyr), and levothyroxine (Euthyrox N 75) were added to three concentrations of fT3 standard solution (100, 50, 5 μM) in the concentration of 50 μM to investigate the selectivity of the biosensor response. Mentioned species were mixed each time with fT3 solutions in a volume ratio of 1:1. DPV analysis was applied in the potential range of 0.3–0.7 V vs. Ag/AgCl.

The stability test of the biosensor was conducted using CV analysis with GCE/Fe_3_O_4_@graphene/Ab/Lac in the presence of 200 µM fT3 for 35 cycles in a potential range of −0.2–1.3 V vs. Ag/AgCl reference electrode with the scan rate of 50 mVs ^−1^.

### 2.6. Real Sample Analysis

The accuracy test for the detection of 200 µM fT3 was performed using DPV in a potential range of 0.3–0.7 V vs. Ag/AgCl in synthetic human serum.

## 3. Results and Discussion

### 3.1. Characterization of Fe_3_O_4_@graphene Nanocomposite

**Figure 3:**
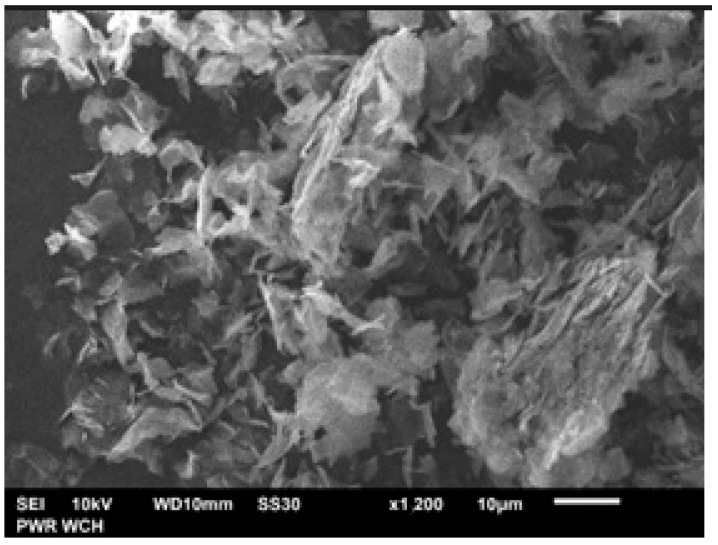
Representative SEM image of the Fe_3_O_4_@graphene nanocomposite.

Applied nanocomposite is commercially available, so the morphology of Fe_3_O_4_@graphene was observed by scanning electron microscopy (SEM). As shown in Figure 3, the microstructure of Fe_3_O_4_@graphene was well-ordered, which implies a good mixing state of graphene and Fe_3_O_4_. This nanocomposite can be used as a modifier in biosensor development, which requires, above all, proper preparation of the electrode for further modification.

### 3.2. Electrochemical Characterization of the Electrode Modification

**Figure 4:**
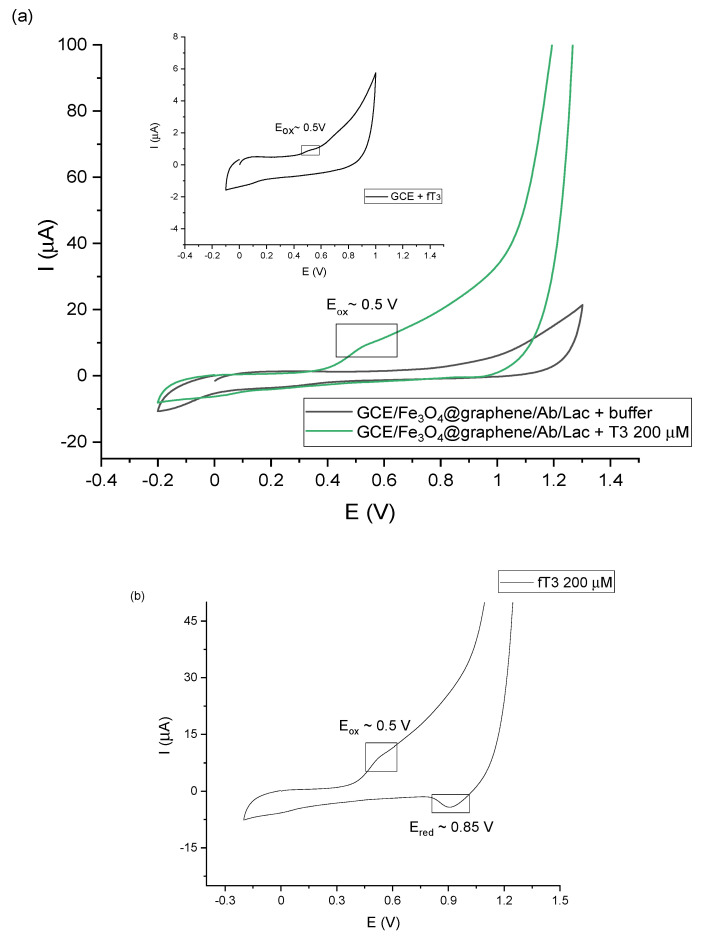
(**a**) Representative CV scans of GCE/Fe_3_O_4_@graphene/Ab/Lac (green line) in the presence of 200 μM fT3, and GCE/Fe_3_O_4_@graphene/Ab/Lac in PBS buffer (black line); inset: CV scan of a bare GCE in the presence of fT3; (**b**) GCE/Fe_3_O_4_@graphene/Ab/Lac in 200 μM fT3; all measurements were performed under applied potential in the range of −0.2–1.3 V, scan rate 50 mV s^−1^, vs. Ag/AgCl.

**Figure 5:**
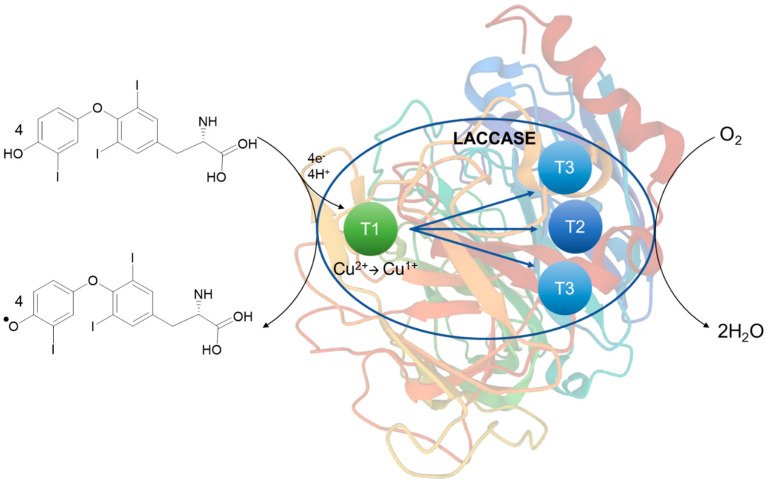
Scheme of the proposed mechanism of the redox enzyme-catalyzed fT3 oxidation.

Taking into account the above-mentioned information, the developed biosensor has been constructed as described in Section 2.3 and allowed measuring the amperometric response obtained from the fT3 hormone. The detection process starts with a specific recognition between the antibody and the analyte. It is important to note that because of the size, an antibody molecule can bind only one enzyme molecule. In this case the steric hindrance can be reduced and the enzyme can catalyze the reaction on a specific substrate while maintaining high activity [42]. According to this, immobilized laccase can catalyze the oxidation of fT3, which can be recorded through electrochemical measurements. Figure 4 presents the cyclic voltammograms of the biosensor response to fT3, as well as the whole redox reaction process. As can be observed in Figure 4a, GCE/Fe_3_O_4_@graphene/Ab/Lac response (green line) was recorded in the presence of 200 μM fT3 (prepared in 0.1 M PBS buffer at pH 7.0) in a potential range −0.2–1.3 V, with a scan rate of 50 mV s^−1^. Moreover, a “background” signal, resulting from 0.1 M PBS buffer, pH 7.0 (black line), was recorded on the voltammogram. The signal corresponding to fT3 with the biosensor showed the current response at approximately 10 µA. In addition, the fT3 signal at the bare GCE was slightly visible (10 times lower—1 µA), which indicates that the bare electrode can oxidize fT3 in a limited way. Used in a biosensor part, an anti-PDIA3 is a major non-nuclear binding protein of the thyroid hormone 3,3′,5-triiodo-l-thyronine. Selected potential range also allows the observation of the entire fT3 reaction process with the use of the biosensor (Figure 4b) occurring in the potential range −0.2–1.3 V, where visible peaks are at 0.5 V (anodic direction) and 0.85 V (cathodic direction). The signal at around 0.5 V corresponds to the oxidation signal of fT3 (enzyme-dependent reaction), and is characteristic for the fT3 oxidation, while the reduction signal may correspond to the one of the laccase active center [43]. Based on Figure 4b, the redox signal visible at 0.85 V can be assigned to the reduction of the laccase active center. Piontek et al. for the first time described why laccase from *T. versicolor* possesses a higher redox potential (around 0.8 V) in comparison with other laccases [44]. In this enzyme, the Cu type 1 atom is 3-fold coordinated and has no axial ligand. Type-1 copper in the laccase is in a planar arrangement, which creates a flat trigonal system by a coordinate bond with nitrogen atoms of the three histidine rings. Instead of methionine, there is usually phenylalanine that does not provide coordinate bonding with Cu type-1, which could result in higher redox potentials [45]. The proposed enzyme-dependent mechanism of the oxidation reaction of fT3, once it is targeted by the antibody, is presented in Figure 5.

### 3.3. Analytical Performance of the Voltammetric Biosensor

For the characterization of the working parameters of the biosensor, DPV was employed. DPV involves applying amplitude potential pulses on a linear ramp potential; a value of a base potential, at which there is no faradaic reaction, is chosen and applied to the electrode [18]. Current is measured before the application of the pulse and at the end, and the current difference is recorded [46]. The biggest advantage of this technique is low capacitive current, which results in high sensitivity, which is why this technique was used for the description of the analytical performance of the described biosensor.

**Figure 6:**
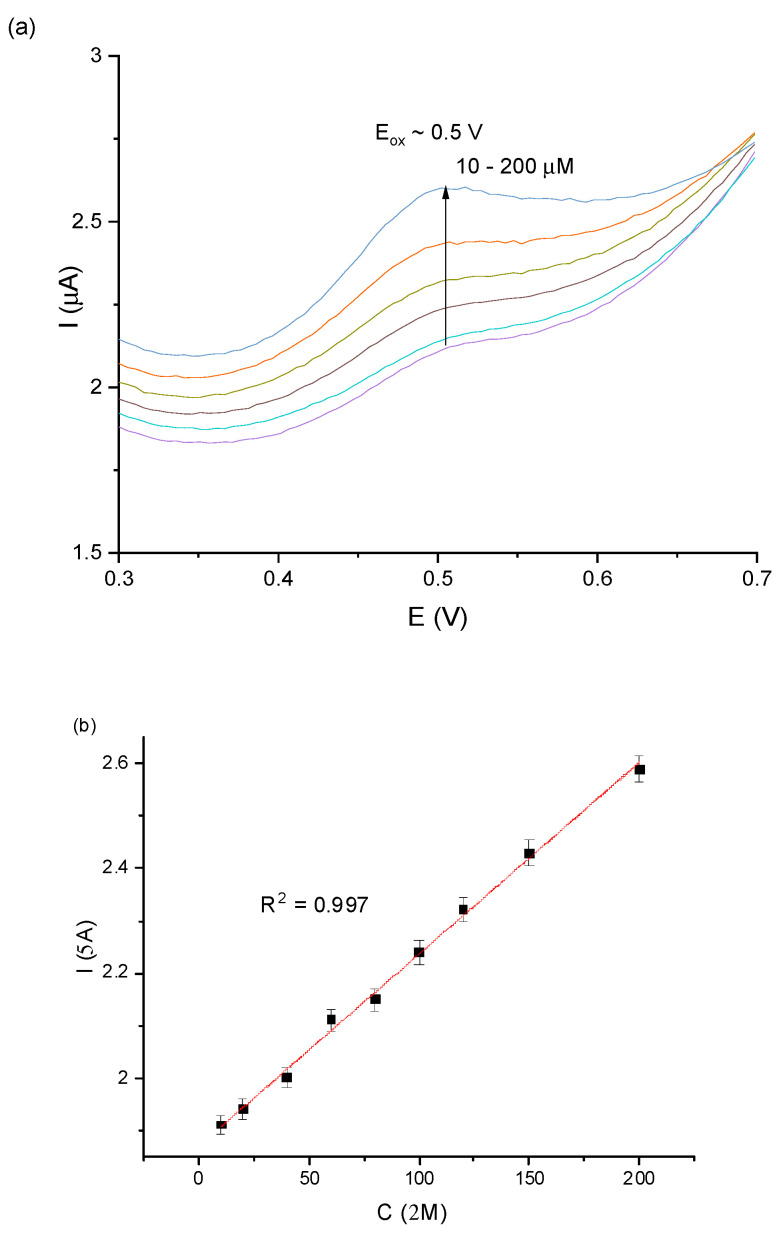
(**a**) Representative DPV scans for different concentrations of fT3 in the range of 10–200 μM vs. Ag/AgCl; (**b**) linear relationship between current and fT3 concentration (10–200 μM).

To evaluate the immunochemical interaction between Anti-PDIA3 Ab and fT3, Fe_3_O_4_@graphene/Ab/Lac modified GCE was exposed to various concentrations of fT3 (in a range of 10–200 µM) in 0.1 PBS, pH 7.0. Figure 6a shows a DPV voltammogram of recorded signals, which precisely correspond to a given fT3 concentration. The observed changes in current increased proportionally with fT3 concentration in a wide range of 10–200 µM. On the current vs. concentration plot, presented in Figure 6b, the linear relationship is presented, based on DPV measurements of the biosensor (GCE/Fe_3_O_4_@graphene/Ab/Lac) for the investigated concentration range with excellent linear response to fT3 (linear coefficient R^2^ = 0.997). Additional parameters for the analytical validation are presented in the Supplementary Materials.

The theoretical limit of detection calculated using Equation S1 (described in the Supplementary Materials) was equal to 27 nM. However, the LOD based on the obtained results is equal to the minimum concentration of the analyte—10 µM—which was determined with the constructed bioanalytical system. Scientific reports regarding the detection of thyroid hormones are limited, which is why comparison of presented value is difficult. However, there are a few reports presenting electrochemical immunosensors for fT3 analysis in the literature. For instance, Chou et al. proposed an electrochemical immunosensor utilizing an electrochemiluminescence technique for fT3 monitoring based on fT3-conjugated, silver nanoparticle-decorated carboxylic graphene oxide (Ag@fGO-FT3) as a carrier, and anti-fT3 antibody-tris(2,2′-bipyridyl) ruthenium (II) (Ru(bpy)_3_^2+^) as a probe. The authors reached an excellent detection limit equal to 0.77 pM and linearity in a range of 15.36 pM–12.29 nM [9]. Another system proposed by Nguyen et al. used electrochemical impedance spectroscopy (EIS) as a detection method for fT3, with a gold electrode modified with gold nanoparticles and an antibody. The group reported fT3 linearity in a range of 153.62 nM–15.36 μM. Moreover, according to Sterling et al. [47], concentration of normal/total T3 (bound and free fractions of T3) in human serum is approximately 3 nM. Diagnostic tests are focused basically on bound T3, and while measurement of free T3 is possible, it may be not reliable and therefore may not be helpful [48]. For patients with thyroid disorders, such as hypothyroidism, fT3 concentration may decrease to a range of even 0.5–1 nM. The obtained detection limit showed that the parameters obtained in this paper are higher in case of a reliable detection system for such patients. However, it can be successfully used in patients suffering from hyperthyroidism or nodular goiters, where the concentration of fT3 is higher than normal, and may reach values much higher than 12 nM [47].

The theoretical limit of quantification (LOQ) for constructed biosensor was determined based on Equation S2 (described in the Supplementary Materials) and was equal to 45.9 nM [49]. The sensitivity of the proposed biosensor was found at 2.8 μA mM^−1^cm^−2^.

### 3.4. Selectivity and Sensitivity

**Figure 7:**
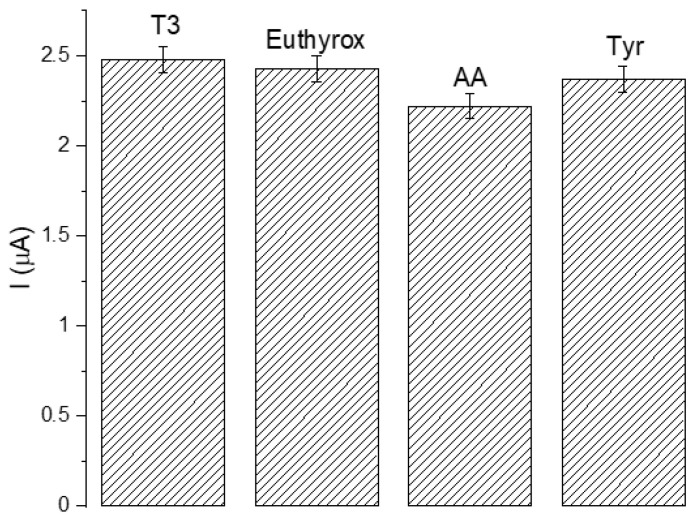
Influence of interfering substances (50 μM) on fT3 detection.

Presented biosensor for fT3 analysis should be suitable for diagnostic purposes. Selectivity is one of the most important parameters of biosensors; this is why to ensure the feasibility of the constructed biosensor, a few species similar in structure and most common in human samples were selected as nontarget compounds to confirm the selectivity of the sensor, under the same conditions (levothyroxine–Euthyrox, ascorbic acid (AA) and tyrosine (Tyr)). Of particular importance was levothyroxine, an isomer of T4, as it is both very similar in structure to fT3, as well as being a drug commonly used in treating thyroid disorders, which is why it is plausible to be found in the samples tested for fT3. All compounds were added (in a concentration of 50 μM) to the investigated fT3 samples at concentrations equal to 1, 50, and 100 μM to test the impact in high excess, equilibrium, and deficiency of them on the measurements. As can be observed in Figure 7, all interfering species have negligible effect on the fT3 detection (average < 4.7%), current response is stronger in fT3 than that of other interfering compounds, which suggests very high selectivity of the presented assay.

**Figure 8:**
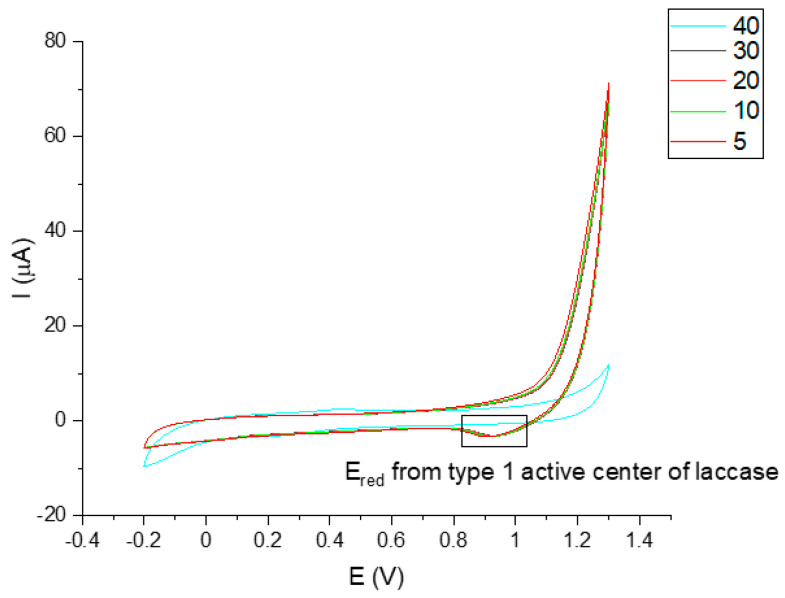
Representative CV scans of GCE/Fe_3_O_4_@graphene/Ab/Lac in the presence of 200 μM fT3 after 21 days. Potential range: −0.2–1.3 V, scan rate: 50 mV s^−1^, vs. Ag/AgCl electrode, 40 cycles.

Long-term stability is an important issue in biosensor fabrication because of the often relatively fast loss of the catalytic activity of biologically active materials. In the presented biosensor, stability was achieved by storing the prepared biosensor at 4 °C (humid conditions) after finishing all previous experiments. The detection of fT3 was then repeated and carried out after storage for 21 days for 40 cycles using the CV technique (potential range: −0.2–1.3 V, scan rate: 50 mV s^−1^, vs. Ag/AgCl electrode) (Figure 8). The average response current retained 89.4% of the initial current after 21 days (40 cycles), which shows that the constructed biosensor exhibited high stability.

### 3.5. Real Sample Analysis

The constructed GCE/Fe_3_O_4_@graphene/Ab/Lac biosensor was also tested for the accuracy of the proposed method, which provides information on the practicability of the described study for future application, for instance, in the medical diagnostic field. A real sample detection ability of the immunoassay was examined as follows: fT3 was dissolved in 10 mL of synthetic human serum to obtain the final concentration of 200 µM of the analyte. The DPV method was applied to record the signal, which was compared to the signal obtained in previous results for 200 µM of fT3 from the linear curve (linear regression equation). As shown in Table 1, the prepared biosensor presented a very good recovery result (calculated as a ratio of the signal detected for fT3 in a buffer to the signal obtained from fT3 in the synthetic human serum (%)), which indicates a possibility of future application in medical diagnostic tests.

## 4. Conclusions

The presented study demonstrates that a biosensing platform based on GCE/Fe_3_O_4_@graphene/Ab/Lac is a good candidate for an biosensing assay. Its focus is on a direct, easy-to-prepare, selective, and sensitive test for the analysis of fT3. Thanks to the proposed analytical method, a quantitative analysis of fT3 is achieved in a wide concentration range of 10–200 μM, with a detection limit equal to 27 nM. The described biosensor is also very specific, sensitive (even in the presence of levothyroxine), and has excellent recovery in synthetic human serum. The proposed assay could, in principle, be adapted as a universal test with good stability for over 30 days. Furthermore, we proposed a possible mechanism of enzyme-based fT3 oxidation by laccase. In comparison to previously mentioned works, our biosensor used commonly available and environmentally friendly materials, an easy technique (DPV) that can also potentially be used in mobile sensor devices, and is characterized by high selectivity, working in a wide linear range and a low detection limit. In our opinion, the presented biosensor for the detection of fT3 has a high potential to be used for an early determination of diseases related to fT3 level disturbances (e.g., hyperthyroidism) after some optimization.

## Figures and Tables

**Table 1:** Results obtained for the detection of fT3 in synthetic human serum.

Concentration of fT3 in a Real Sample (µM)	C_detected_ (µM)	Recovery (%)	RSD
200.00	193.00	96.5	±0.67

## Supplementary Materials

The following supporting information can be downloaded at: https://www.mdpi.com/article/10.3390/s23020602/s1, Table S1: Analytical parameters of the calibration curve of GCE/Fe_3_O_4_@graphene/Ab/Lac with DPV technique.
