# Supplementary material for: A Novel Strategy for Selective Thyroid Hormone Determination Based on an Electrochemical Biosensor with Graphene Nanocomposite
Source: Sensors (Basel). 2023 Jan 5;23(2):602. doi: 10.3390/s23020602 (PMC9861372; doi:10.3390/s23020602)
Supplement: Supplementary file 1 [file sensors-23-00602-s001.zip › sensors-2080070-supplementary.pdf]

A Novel Strategy for Selective Thyroid Hormone Determination Based on an Electrochemical Biosensor with Graphene Nanocomposite

Sylwia Baluta[1], Marta Romaniec[1], Kinga Halicka-Stępień[1], Michalina Alicka[2], Aleksandra Pieła[1], Katarzyna Pala[2], Joanna Cabaj[1]

[1] Faculty of Chemistry, Wrocław University of Science and Technology, Wybrzeże Wyspiańskiego 27, 50-370 Wrocław, Poland
[2] Food4Future Technologies Sp. z o. o., ul. Tarasa Szewczenki 24, 51-351 Wrocław
* Correspondence: joanna.cabaj@pwr.edu.pl

Supplementary Materials

Table S1. Analytical parameters of the calibration curve of GCE/Fe_3O_4@graphene/Ab/Lac with DPV technique.

Linear range	LOD	LOQ	R^2	Slope	SD of slope	Intercept	SD of intercept
10 – 200 μM	27 nM	45.9 nM	0.997	0.028	0.0011	1.28	0.0046

Equation S1. Limit of Detection

The limit of detection (LOD) for described method was calculated as shown in equation S1:

$$LOD = 3.29\ \sigma_B/b, \quad (S1)$$

where σ_B is the standard deviation of the population of blank responses and b is the slope of the regression line.

Calculated LOD was equal to 27 nM.

Equation S2. Limit of Quantification

Theoretical limit of quantification (LOQ) for constructed biosensor was determined based on equation S2:

$$LOQ = 5\ \sigma_B/b, \quad (S2)$$

where σ_B is the standard deviation of the population of blank responses and b is the slope of the regression line, and was equal to 45.9 nM.
